# Patient Lung Cancer Screening Decisions and Environmental and Psychosocial Factors

**DOI:** 10.1001/jamanetworkopen.2024.12880

**Published:** 2024-05-31

**Authors:** Jennifer Richmond, Jessica R. Fernandez, Kemberlee Bonnet, Ashley Sellers, David G. Schlundt, Allana T. Forde, Consuelo H. Wilkins, Melinda C. Aldrich

**Affiliations:** 1 Division of Public Health Sciences, Department of Social Sciences and Health Policy, Wake Forest University School of Medicine, Winston-Salem, North Carolina; 2 Division of Intramural Research, National Institute on Minority Health and Health Disparities, National Institutes of Health, Bethesda, Maryland; 3 Department of Psychology, Vanderbilt University, Nashville, Tennessee; 4 Qualitative Research Core, Vanderbilt University Medical Center, Nashville, Tennessee; 5 Division of Geriatric Medicine, Department of Medicine, Vanderbilt University Medical Center, Nashville, Tennessee; 6 Division of Genetic Medicine, Department of Medicine, Vanderbilt University Medical Center, Nashville, Tennessee; 7 Department of Biomedical Informatics, Vanderbilt University Medical Center, Nashville, Tennessee; 8 Department of Thoracic Surgery, Vanderbilt University Medical Center, Nashville, Tennessee; 9 NORC at the University of Chicago, Bethesda, Maryland

## Abstract

**Importance:**

Screening for lung cancer using low-dose computed tomography is associated with reduced lung cancer–specific mortality, but uptake is low in the US; understanding how patients make decisions to engage with lung cancer screening is critical for increasing uptake. Prior research has focused on individual-level psychosocial factors, but environmental factors (eg, historical contexts that include experiencing racism) and modifying factors—those that can be changed to make it easier or harder to undergo screening—also likely affect screening decisions.

**Objective:**

To investigate environmental, psychosocial, and modifying factors influencing lung cancer screening decision-making and develop a conceptual framework depicting relationships between these factors.

**Design, Setting, and Participants:**

This multimethod qualitative study was conducted from December 2021 to June 2022 using virtual semistructured interviews and 4 focus groups (3-4 participants per group). All participants met US Preventive Services Task Force eligibility criteria for lung cancer screening (ie, age 50-80 years, at least a 20 pack-year smoking history, and either currently smoke or quit within the past 15 years). Screening-eligible US participants were recruited using an online panel.

**Main Outcomes and Measures:**

Key factors influencing screening decisions (eg, knowledge, beliefs, barriers, and facilitators) were the main outcome. A theory-informed, iterative inductive-deductive approach was applied to analyze data and develop a conceptual framework summarizing results.

**Results:**

Among 34 total participants (interviews, 20 [59%]; focus groups, 14 [41%]), mean (SD) age was 59.1 (4.8) years and 20 (59%) identified as female. Half had a household income below $20 000 (17 [50%]). Participants emphasized historical and present-day racism as critical factors contributing to mistrust of health care practitioners and avoidance of medical procedures like screening. Participants reported that other factors, such as public transportation availability, also influenced decisions. Additionally, participants described psychosocial processes involved in decisions, such as perceived screening benefits, lung cancer risk appraisal, and fear of a cancer diagnosis or harmful encounters with practitioners. In addition, participants identified modifying factors (eg, insurance coverage) that could make receiving screening easier or harder.

**Conclusions and Relevance:**

In this qualitative study of patient lung cancer screening decisions, environmental, psychosocial, and modifying factors influenced screening decisions. The findings suggest that systems-level interventions, such as those that help practitioners understand and discuss patients’ prior negative health care experiences, are needed to promote effective screening decision-making.

## Key Points

**Question:**

Do environmental, psychosocial, and modifying factors influence patient decisions to undergo screening for lung cancer?

**Findings:**

In this qualitative study consisting of interviews and focus groups with 34 individuals eligible for lung cancer screening, participants emphasized historical and present-day racism as a critical factor contributing to mistrust of health care practitioners and avoidance of cancer screening. Participants also identified key psychosocial and modifying factors involved in lung cancer screening decisions, such as health insurance coverage and fear of a cancer diagnosis.

**Meaning:**

The findings suggest that systems-level interventions are needed to address environmental, psychosocial, and modifying barriers to effective lung cancer screening decision-making.

## Teaser

This qualitative study investigates environmental, psychosocial, and modifying factors in lung cancer screening decisions among US patients meeting screening eligibility criteria.

## Introduction

Lung cancer is the leading cause of cancer death in the US.^1^ Screening for lung cancer using low-dose computed tomography (LDCT) of the chest is associated with reduced lung cancer–specific mortality because it improves the chances of an early-stage diagnosis.^2,3^ The US Preventive Services Task Force (USPSTF) recommends annual lung cancer screening for adults aged 50 to 80 years with at least a 20 pack-year smoking history and who currently smoke or have quit smoking within the past 15 years.^4^ Pack-years are calculated by multiplying the number of cigarette packs that someone has smoked per day by the number of years they have smoked.^5^ Lung cancer screening uptake is low, ranging from 5% to 21% among eligible individuals in the US.^6,7,8,9^ Screening receipt is even lower among US populations that bear a disproportionate burden of lung cancer, such as Black individuals.^10,11,12,13,14,15^ In the US, Black individuals are 16% less likely than White individuals to survive 5 years after a lung cancer diagnosis but are also less likely than White individuals to receive LDCT.^13,16,17^

Understanding the lung cancer screening decision-making process is a necessary component of increasing uptake. This process is especially important for lung cancer screening because the Centers for Medicare & Medicaid Services requires that Medicare beneficiaries have a documented shared decision-making visit before undergoing LDCT to understand potential benefits and harms.^18^ Therefore, it is vital that health care practitioners understand the factors that patients consider when making screening decisions so they can offer appropriate information during shared decision-making visits.

Prior research has focused on individual-level psychosocial factors involved in LDCT decisions (eg, perceived lung cancer risk).^19,20,21^ Yet, environmental factors, such as availability of public transportation, may also impact cancer screening decisions.^19,22^ Furthermore, the US history of racism, such as unethical medical experimentation on Black people, has also affected engagement with the health care system.^23,24,25^ Discussions of racism are largely absent from the lung cancer screening literature, though.^19,20,21,26,27^ One notable study created a conceptual model of lung cancer screening participation that highlights contributing social and environmental factors.^26^ That model focuses on media exposure and influences of family and friends as social or environmental factors, creating opportunities to investigate racism and other environmental factors.^26,28^ Finally, modifying factors—factors that can be changed to make it easier or harder to receive screening—are understudied in screening decision-making. To address these knowledge gaps, we examined environmental, psychosocial, and modifying factors influencing lung cancer screening decision-making and developed a conceptual framework to summarize relationships between these factors.

## Methods

### Participant Recruitment

This multimethod qualitative study consisted of virtual semistructured interviews and 4 focus groups (3-4 participants per group) with participants throughout the US. We conducted both interviews and focus groups to diversify the information elicited (eg, information on a wide range of participant experiences from focus groups and detailed information on these experiences from interviews).^29^ The sample size was informed by recommendations for conducting online qualitative research and the number of interviews or focus groups needed to reach saturation.^30,31,32,33^ The Vanderbilt University institutional review board deemed this study exempt per 45 CFR §46.^34^ Participants provided written informed consent. This study followed the Consolidated Criteria for Reporting Qualitative Research (COREQ) reporting guideline.

We used a purposive sampling approach to identify participants with insights relevant to lung cancer screening.^35^ Participants were eligible if they met USPSTF eligibility criteria for lung cancer screening (age 50 to 80 years, at least a 20 pack-year smoking history, and either currently smoke or quit within the past 15 years). We also recruited a study population in which about half of participants self-identified as Black, a group that has been disproportionately affected by lung cancer.^1^ The race and ethnicity categories included in the study were Asian, Black or African American, Black and American Indian, Black and White, Hispanic or Latino, and White and were ascertained by self-report.

We partnered with CloudResearch, an organization specializing in using online platforms for research recruitment, to identify participants for a 1-time interview or focus group.^36^ CloudResearch used its Amazon Mechanical Turk (MTurk) Approved List and Prime Panels platforms to facilitate recruitment.^37,38,39,40,41^ CloudResearch’s MTurk Approved List is a list of MTurk participants who meet quality metrics (eg, no history of using technology to automatically complete surveys).^41^ MTurk has a smaller pool of participants than Prime Panels, but unlike Prime Panels, it supports real-time recruitment, by which researchers post a study and eligible participants can join the interview within minutes. We used MTurk to recruit in real time until we were unable to reach additional participants (after 9 interviews). We used Prime Panels to recruit for the remaining interviews and focus groups. CloudResearch had access to participant demographic information, which was leveraged to purposively recruit a sample in which about half of participants self-identified as Black.

To assess study eligibility, potential participants completed an online REDCap survey (eAppendix 1 in Supplement 1).^42^ This survey included questions about demographic characteristics, smoking history, and prior screening receipt. Participants who met eligibility criteria received additional study information and reviewed an informed consent form, which included permission to record sessions and publish deidentified findings. Because MTurk supports real-time recruitment, these participants received the link to join the video study after consenting and were permitted to immediately begin the study. Prime Panels has a lag between study posting and when the study is visible to participants. Therefore, Prime Panels participants received information about upcoming interview or focus group times and, after consenting, signed up for a session. No participants dropped out of the study after consenting. All data were stored on secure servers.

### Interviews and Focus Groups

We collaborated with a local community advisory board^43^ composed of 8 community members with various cancer diagnoses, tobacco use experiences, and racial and ethnic backgrounds and a history of collaborating with the study team (J.R., M.C.A.) on lung cancer research to develop semistructured interview and focus group guides (presented in eAppendixes 2 and 3 in Supplement 1). The guides included questions about factors influencing lung cancer screening decisions (eg, knowledge, beliefs, and environmental barriers). Staff with qualitative methods expertise and training at the Vanderbilt University Medical Center Qualitative Research Core^44^ (K.B.) conducted interviews and focus groups using a videoconferencing platform. The principal investigator (J.R.), who has doctoral-level training in qualitative methods and has conducted qualitative research for over a decade, observed all sessions, presented the study purpose to participants, and answered questions as needed. No participants had existing relationships with the researchers. The interviewer and principal investigator wrote field notes and regularly debriefed after interviews and focus groups to record insights emerging from the data and to reflexively consider how context (eg, the interviewer’s and principal investigator’s backgrounds) influenced the research.^45^ Furthermore, the principal investigator regularly reflected to examine how her background, experiences with cancer, and identity as a multiracial Black woman motivated her to pursue this study.

Interviews lasted approximately 30 to 60 minutes, and focus groups lasted approximately 90 minutes. Interviews and focus groups were audiorecorded and professionally transcribed. Transcripts and study data were not returned to study participants for comment to avoid creating identifiable links between participants and their data. During data collection and analysis, we reviewed field notes and transcripts to examine codes arising in the data and assess saturation.^46^ After 20 interviews and 4 focus groups, no new insights were emerging from the data. All sessions occurred between December 2021 and June 2022.

### Data Analysis

The COREQ guideline informed data coding and analysis.^47^ We developed an initial hierarchical coding system and codebook and iteratively refined it by using the interview and focus group guides and a preliminary review of transcripts.^48^ Examples of major coding categories include health and social history, emotions and feelings, attitudes and beliefs, coping, encounters with health care practitioners, LDCT decision-making, screening barriers and facilitators, and societal factors relevant to screening.

To establish intercoder reliability, 2 experienced coders independently coded 1 interview and 1 focus group transcript. We compared coding and resolved all discrepancies. After reaching consensus in use of the coding system, the 2 coders divided and independently coded the remaining transcripts. Microsoft 365 Excel, version 2402, and SPSS, version 28.0 (IBM Corp), were used to manage data analysis (eg, to sort files by code and run cross-tabulations to examine code co-occurrences).^49,50^

We used an iterative inductive-deductive approach to identify higher-order themes.^51,52^ The goal of this inductive-deductive approach was to develop a conceptual framework deductively informed by established theories while inductively integrating details from the qualitative data. Deductively, the analysis was informed by Social Cognitive Theory, which posits that behavior is influenced by interactions of personal, environmental, and behavioral factors.^53,54^ We were also guided by the Health Belief Model, a theory positing that health decisions are informed by several factors, such as perceived benefits and risks.^55^ These theories have been widely used for decades to explain health behavior and, when combined, may elucidate the influence of psychosocial and environmental factors on health behaviors.^55,56,57^ Inductively, the framework’s content was derived from the qualitative data.

## Results

### Participant Characteristics

A total of 34 individuals participated in this study (20 [59%] in interviews and 14 [41%] in focus groups). Mean (SD) age was 59.1 (4.8) years, and mean (SD) smoking history was 39.3 (21.2) pack-years. Twenty participants (59%) identified as female and 14 (41%) as male. Overall, 2 (6%) identified as Asian, 16 (47%) as Black or African American, 1 (3%) as Black and American Indian, 1 (3%) as Black and White, 1 (3%) as Hispanic or Latino, and 13 (38%) as White. Half of participants (17 [50%]) had an annual household income of less than $20 000. Most participants had health insurance (31 [91%]), lived in the southern US census region (22 [65%]), and had never been screened for lung cancer (27 [79%]). Table 1 presents participant sociodemographic characteristics.

**Table 1.** Characteristics of Interview and Focus Group Participants

Characteristics	Participants^a^		
	Interviews (n = 20)	Focus groups (K = 4, n = 14)	Overall (N = 34)
Age, mean (SD), y	59.8 (4.4)	58.1 (5.4)	59.1 (4.8)
Self-reported gender			
Female	12 (60)	8 (57)	20 (59)
Male	8 (40)	6 (43)	14 (41)
Race and ethnicity			
Asian	1 (5)	1 (7)	2 (6)
Black or African American	10 (50)	6 (43)	16 (47)
Black and American Indian	0	1 (7)	1 (3)
Black and White	1 (5)	0	1 (3)
Hispanic or Latino	0	1 (7)	1 (3)
White	8 (40)	5 (36)	13 (38)
Educational level			
High school or less	6 (30)	2 (14)	8 (24)
Some college or associate’s degree	10 (50)	10 (71)	20 (59)
Bachelor’s degree or higher	4 (20)	2 (14)	6 (18)
Annual income, $			
<10 000	4 (20)	3 (21)	7 (21)
10 000-19 999	4 (20)	6 (43)	10 (29)
20 000-29 999	3 (15)	3 (21)	6 (18)
30 000-59 999	5 (25)	2 (14)	7 (21)
≥60 000	4 (20)	0	4 (12)
Marital status			
Never married	5 (25)	4 (29)	9 (26)
Married or living with a partner	2 (10)	3 (21)	5 (15)
Separated or divorced	10 (50)	5 (36)	15 (44)
Widowed	3 (15)	1 (7)	4 (12)
Missing	0	1 (7)	1 (3)
Health insurance type			
Private insurance	7 (35)	3 (21)	10 (29)
Medicare	6 (30)	4 (29)	10 (29)
Medicaid	6 (30)	4 (29)	10 (29)
TRICARE or military insurance	0	1 (7)	1 (3)
None	1 (5)	2 (14)	3 (9)
Employment status			
Employed	8 (40)	2 (14)	10 (29)
Unemployed	1 (5)	4 (29)	5 (15)
Retired	3 (15)	4 (29)	7 (21)
Unable to work	8 (40)	4 (29)	12 (35)
US census region			
Northeast	3 (15)	3 (21)	6 (18)
Midwest	2 (10)	1 (7)	3 (9)
South	13 (65)	9 (64)	22 (65)
West	2 (10)	1 (7)	3 (9)
Current smoker			
Yes	19 (95)	14 (100)	33 (97)
No or former smoker	1 (5)	0	1 (3)
Pack-years smoked in lifetime, mean (SD)	42.5 (25.9)	34.8 (11.2)	39.3 (21.2)
Ever received lung screening			
Yes	4 (20)	3 (21)	7 (21)
No	16 (80)	11 (79)	27 (79)

^a^ Data are presented as number (percentage) of participants unless otherwise indicated.

### Conceptual Framework Overview

The Figure presents the conceptual framework resulting from our analysis. On the left side of the framework, circles representing psychosocial processes (eg, risk appraisal) interact with aspects of the environment (eg, historical context) to influence screening decisions. The middle of the framework presents arrows representing modifying factors (eg, insurance coverage) that can make it easier or harder to receive screening. In the following sections, we describe each element of the framework. Table 2 presents illustrative participant quotes from each framework element.

**Figure.**
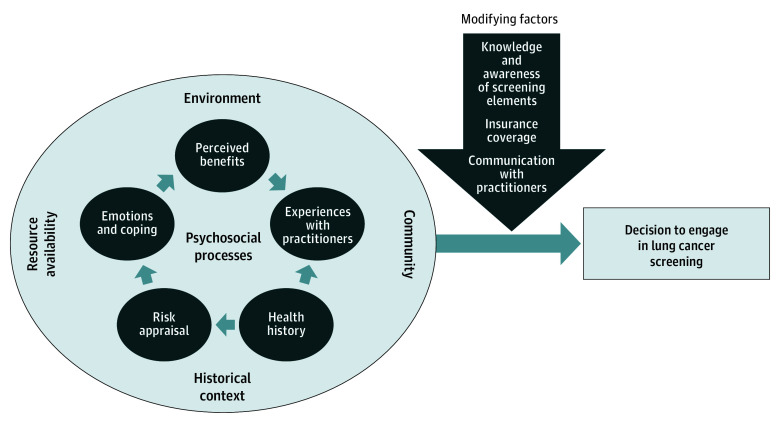
Environmental, Psychosocial, and Modifying Factors Influencing Decisions to Engage in Lung Cancer Screening

**Table 2.** Key Elements and Illustrative Quotes From Lung Cancer Screening Decisions Conceptual Framework

Framework element	Illustrative quotes
**Environment**	
Historical context	“Yeah. Well, there’s some sort of mistrust. In the Black community, in the African American community, because of slavery, there’s a mistrust of police officers. There’s a mistrust of doctors. A Black person would rather go to a Black doctor than a Caucasian doctor. You know what I’m saying? Because the familiarity. And a lot of that distrust comes from racism and it trickles down. It trickles down to other areas like police enforcement. Black people would rather settle things on their own amongst their self, as opposed to calling the police. That’s like the last resort. You know what I’m saying?” (focus group 2, participant 4, never screened) “…There is mistrust among the Black community when it comes to medical care. Especially with Black men. Because of previous experience, many feel that the best medical care will not be provided to us.” (focus group 1, participant 3, never screened) “They call it blue ball. I don’t know if it’s a real name for it, but that’s just something that Black people call it. They say they blue ball them, the blue ball. And so they just inject them with so much morphine that they pass away.” (interview 10, never screened)
Community	“I don’t know if that’s a old stigmatism in my community or in my culture, but a lot of people, ever since I was little, if you don’t have a gunshot wound or you’re missing a limb, they’re not going to the doctor. So, yeah, you really got to be seriously, seriously sick…” (interview 20, never screened) “…Keeping things in the family. I think [my aunt is] sick with cancer right now, to tell you the truth. But it’ll take a act of God for anybody in that family to tell me. I just have to assume it but I wouldn’t speak it out loud. So they still doing it too. My family’s still doing it too. That serious diseases, they battle it within their own family and they keep it to they self. They don’t discuss it on the job. They don’t discuss it in the church. They don’t discuss with their friends. Everybody have a right to do as they feel as long as they’re not harming someone else.” (interview 19, never screened) “Someone also spoke about a witch doctor and Obeah and voodoo. I came from a family that’s with that type of culture. I remember one time I was playing with an old shopping cart and somebody was pushing me on it. The shopping cart flipped over. My leg got caught in and I broke my ankle. And my grandmother didn’t take me to the hospital. My grandmother went and she got some bushes from her garden. She boiled it and she took the leaves out and she wrapped it on my ankle and it stayed on my ankle. I couldn’t walk around much, but within 2, 3 weeks I was running around again like it never happened…” (focus group 2, participant 4, never screened)
Resource availability	“It shouldn’t be about if you can afford it. That’s the whole reason why the health system is so messed up, because it’s all about money. It’s not about the individual. It’s about money.” (focus group 2, participant 1, screened) “Yeah. I mean, my mother’s on oxygen now and she feels like a burden because she doesn’t have her own car and then to lug her around and this tank of air, and then to get it in and out of the car and all the cords hanging, like she feels like a bother. But she’s not a bother. She’s hard on herself about what she’s created.” (interview 8, never screened) “I think transportation would be an issue for a lot of people and hopefully they have employers who are allowing them to take off for an appointment or a follow-up of the cost associated with going to an out of state or an out of area hospital. We have a university hospital that’s maybe an hour and a half away. So there is time and cost associated with it.” (interview 9, screened)
**Psychosocial processes**	
Perceived benefits	“I would say it would be the importance of getting screened. If catching something early might save your life, I think that is really useful.” (interview 3, screened) “…I just think it’s good for both men and women, it keeps you abreast on what’s going on with you as far as health wise. And like you said earlier, it’ll help you take anything early. And it would also help the doctor. It would help you to seek early care.” (interview 13, scheduled for first screening) “…I dreaded hearing that bad news and it might have affected me earlier to not really pushing, even though I was concern, maybe I was like what’s the point? If they find it, it’s not good for me. If they don’t, then I’m going to continue to do what I’m doing, even though I’m going to stop smoking for one day. But I think it’s probably like other diseases, people don’t want to kind of, they’re at the point if they find it’s over anyway, I might as well be enjoying my life and not knowing, which is not good…” (interview 4, screened)
Experiences with practitioners	“It really, it depends on whatever physician I happen to be seeing. If they establish some rapport with me, that makes a huge difference. If they’re somewhat aloof and indifferent and don’t want to hear my input, then that kind of leaves a negative opinion of them pretty much. But that only happened once I think, so.” (interview 2, never screened) “I had my second daughter…and that was the hardest time ever. I felt everything because I had a C-section and they did not numb me. I told them that I was feeling it and they didn’t believe me when they were cutting me…. So they ended up just strapping me down, because I told them, ‘I feel this. You’re hurting me.’ They just strapped me down and continued.” (interview 10, never screened) “Yes. In California. My orthopedic surgeon. I had a fractured hip and it set wrong and he wouldn’t believe it and his partner actually said to me, I was on Medicaid at the time. He said, ‘We only get paid $16 an hour to see you people. We pay our secretaries more than that.’ It’s the most disgusting thing I’ve ever heard…” (interview 7, never screened)
Health history	“To be honest with you I don’t think about it. I have vascular disease and I think that’s going to get me before any cancer does…” (interview 7, never screened) “…I’m still learning how to handle my stress now, but then I get diagnosed with diabetes type 2 in 2017. I passed out…. I wake up 7 days later in ICU trying to figure out what the heck is going on. And I couldn’t see. And that’s when they told me, ‘You’ve got diabetes.’ My sugar was like 2000, believe it or not. So technically I’m not supposed to be here. So God has got his hand on me for a specific reason...lung cancer screening, it’s imperative…. You could be at stage 4 and not even know it, because like he said, cancer, like congestive heart failure, like diabetes, they are silent creepers. They’re silent killers. You don’t know what’s going on with your body until you actually go to a doctor.” (focus group 2, participant 3, never screened) “I mean, I have COPD and I have asthma too, but it’s just when you get a feeling that something’s not right. And I begged the doctor. I want to be screened because I have lung cancer that runs in the family and I just want to make sure that’s not what it is…” (focus group 3, participant 1, never screened)
Risk appraisal	“Because my lifestyle as far as the smoking is concerned has not been remotely healthy. I would rather find out early if I’ve got something and cure it than wait until it’s too far advanced and they’ll say, ‘Well, here. Breathe into this for the rest of your life and sit in a wheelchair.’ I’d rather find out now and have a good chance of getting cured or at least making my remaining life a bit more comfortable than waiting.” (interview 7, never screened) “I’m a smoker, so I know I’m at risk. I’m in the risk category. That’s why you do it. Not because you’re feeling bad or sick or anything like that, hopefully it’s because it’s a screening. If you’re feeling sick and it’s related to your lungs, then it’s already farther along and the farther along it is, the harder it’s to deal with. You don’t have to be a smoker to get it because people get lung cancer. If you’re a smoker and you get lung cancer, you more likely.” (focus group 4, participant 3, never screened) “You know what? That’s funny you say that because I’m still smoking 10-plus cigarettes a day, and I know the health risk, but it doesn’t bother me. I don’t know. Maybe because I’m very active. My wife has had a stroke, so I’ve been her caregiver since back in 2014, and plus, I’m disabled, so I basically be on the go.” (interview 20, never screened)
Emotions and coping	“Part of it could be fear of actually discovering something. I don’t want to get lung cancer screening because I might find out I’ve got lung cancer. Sort of sticking your head in the sand…. I’d rather not know.” (interview 7, never screened) “…If anybody have some type of mistrust, if they feel like their doctor is not doing enough or like I said, being messy in their practices, then I wouldn’t go, and I wouldn’t advise anybody else to go…” (interview 13, scheduled for first screening)
**Modifying factors**	
Knowledge and awareness of screening elements	“Oh, it’s always a plus to know that it’s noninvasive. There’s no needles, there’s no pain. That’s a biggie. Most people are afraid of pain. And just let them know how quick it is, like it only takes 2 minutes.” (interview 12, never screened) “I mean, can you do the test on your own? Go there and drive home by yourself. That’s a big thing. It looks like for me, my support is 200 miles away…. So I got very little support up here. So that would be a big thing for me. Do I need somebody to pick me up after the test? Like colonoscopy you do, and operations you do. You know what I’m saying? That’s a big deal for me. I got some guys I work with. I’m not going to ask them to take off work to pick me up for a test. It’s not going to happen.” (focus group 3, participant 2, never screened)
Insurance coverage	“No, they don’t think about it. They’re more concerned with getting on with their lives, with keeping a roof over their heads, that kind of thing. They’ll go to the doctor if they have to but the vast majority don’t go for preventative visits. A lot of that comes down to money as well and quite a few people I know, they’re in between jobs so they don’t have any insurance, so they won’t go to the doctor even if they’re quite ill.” (interview 7, never screened) “The only thing I think is, you have to deem it medically necessary, even with the insurance. And a lot of times, you’re in a real bad position by the time they get around to screening you for something.” (focus group 3, participant 3, never screened)
Communication with practitioners	“It was my doctor. I went to him because I was having problems with my stomach and he scheduled me all these tests and he asked me, he said, ‘Are you a smoker?’ And I said, ‘Yes, I smoke cigarettes.’ So he said, ‘Well, you need to get this test done and they’ll send me the results and we’ll sit down and we’ll talk about it.’ I said, ‘Okay doc…’” (focus group 2, participant 1, screened) “Yes. The benefits of having the screening, because with me, my doctor, he explained it to the T and he said, ‘Now, you go and evaluate. Is this something that you think you want to do? I understand no one in your family has had lung cancer, but you do have breast cancer and acute myeloid leukemia,’ which is another type of cancer. He say, ‘You never know. Let’s do this and see. Because if so, then we’ll catch it early.’ And the only thing I could think about when he said that was my 18-year-old nephew who had acute myeloid leukemia.” (interview 14, screened) “Well, she said that because I was a smoker that this test is available. And fortunately I have insurance. I didn’t know if there was any additional cost for that, which at first was a concern, because sometimes the insurance companies don’t pay for elective things. But she reassured me that it would be covered through my insurance. She called and got that information for me. She explained to me what the process was of going to the hospital and that, and it was reassuring to me.” (interview 9, screened) “It doesn’t feel good at all because it’s like they’re not concerned about me, my doctors. And I have 2 doctors and neither…mentioned a lung screening test, but mammogram and a colonoscopy yearly, yearly basis, basic. My blood pressure, they check that.” (interview 17, never screened)

Abbreviations: COPD, chronic obstructive pulmonary disease; ICU, intensive care unit.

### Qualitative Findings for Elements of the Conceptual Framework

#### Environment

##### Historical Context

Knowledge of and beliefs about the mistreatment of historically and contemporarily marginalized populations in the US influenced several participants’ decisions to seek lung cancer screening. Several participants, most of whom identified as Black, described how experiences of racism cause mistrust and health care avoidance, particularly in Black communities. One participant explained, “In the Black community..., because of slavery, there’s a mistrust of police officers. There’s a mistrust of doctors... And a lot of that distrust comes from racism and it trickles down.” Another participant elaborated that, “there is mistrust among the Black community when it comes to medical care... Because of previous experience, many feel that the best medical care will not be provided to us.” Participants also described community knowledge of health care atrocities that cause avoidance of medical care: “They call it blue ball...that’s just something that Black people call it... And so they just inject them with so much morphine that they pass away.”

##### Community

Participants also described community cultures in which personal health is not discussed with others, medical visits are reserved for emergencies, and preventive care is avoided. For example, 1 participant explained, “I guess it’s an older generation thing. You didn’t talk about stuff.” Another participant elaborated that in their community, “if you don’t have a gunshot wound or you’re missing a limb, they’re not going to the doctor.” To avoid medical care, some participants described using local healing traditions that had been passed down in their communities.

##### Resource Availability

Participants identified key resources required to engage in lung cancer screening. Most frequently, these resources included income stability and access to transportation. Participants expressed frustration with the US health care system, “because it’s all about money. It’s not about the individual.” Accordingly, participants described how lung cancer screening is inaccessible to those who cannot afford or access it.

#### Psychosocial Processes

##### Perceived Benefits

Most commonly, participants described screening as an important tool to catch disease early and improve treatment outcomes. Yet, others struggled to describe any benefits and questioned whether screening would benefit them. These participants often explained that they would prefer not to know if they had cancer. One participant noted, “What’s the point [of screening]? If they find it [lung cancer], it’s not good for me. If they don’t, then I’m going to continue to do what I’m doing, even though I’m going to stop smoking for one day.”

##### Experiences With Practitioners

Participants largely described the importance of health care practitioner rapport and communication to facilitate trust, which could motivate screening decisions. However, participants frequently described negative, harmful practitioner experiences that diminished their willingness to seek future care. These participants described misdiagnoses, being dismissed when describing pain, and being rushed out of medical offices. One participant described extreme pain that practitioners ignored during a cesarean delivery: “I told them that I was feeling it and they didn’t believe me when they were cutting me... So they ended up just strapping me down, because I told them, ‘I feel this. You’re hurting me.’ They just strapped me down and continued.” Participants, particularly those who had Medicaid or no health insurance at some time in their lives, also described being dismissed due to their insurance status.

##### Health History

Participants often had complex health histories and were diagnosed with comorbidities that impacted screening decisions. These participants expressed exhaustion with frequent medical procedures and explained that other health conditions often took precedence and made it hard to determine screening benefits. One participant described that, “I have vascular disease and I think that’s going to get me before any cancer does.” Conversely, awareness of their health status motivated other participants to engage in preventive care before their health worsened.

##### Risk Appraisal

Participants had differing perceptions of their personal lung cancer risk. Most participants noted the health risks associated with smoking as a factor that could motivate decisions to undergo screening. One participant explained, “I’m a smoker, so I know I’m at risk... That’s why you [get screened].” Other participants expressed that health-promoting habits (eg, physical activity) might mitigate their lung cancer risk: “I’m still smoking 10-plus cigarettes a day, and I know the health risk, but it doesn’t bother me... Maybe because I’m very active.”

##### Emotions and Coping

Participants identified “fear of actually discovering [lung cancer]” as a major barrier to screening. Participants also described fear arising from hearing stories about people who were healthy before a routine health care practitioner visit rapidly led to a cancer diagnosis and death. For example, 1 participant described knowing a healthy man who was diagnosed with cancer after undergoing a routine physical examination: “But I think that if he had...not had that physical, I think he would’ve just kept on living. It looked like the moment they told him he had it, he just started going downhill that day.” Participants who mistrusted practitioners also reported fearing future harm from the health care system. To cope with these fears, participants described avoiding medical practitioners.

#### Modifying Factors

##### Knowledge and Awareness of Screening Elements

Participants raised several questions about screening elements, reflecting how screening knowledge and awareness could affect decisions. The most common question was whether LDCT is invasive or painful. Other participants wondered how much time the procedure would take, how far they would have to travel, and how accurate the test is. Many participants also wondered whether they would need someone to pick them up after the procedure.

##### Insurance Coverage

Participants often expressed interest in being screened but wanted assurance that it would be covered by health insurance. Without insurance coverage, screening was described as inaccessible due to out-of-pocket costs. Although most participants currently had health insurance, they still expressed uncertainty about costs and worried their insurance carrier would not “deem it medically necessary” to avoid paying for screening.

##### Communication With Practitioners

For participants who had received lung cancer screening, direct practitioner communication was a key facilitator motivating their decision. One of these participants described how their doctor “explained it to the T and he said, ‘Now, you go and evaluate. Is this something that you think you want to do?... Because if so, then we’ll catch it early.’” Conversely, participants who had not been screened explained that their practitioners never mentioned it, which was often frustrating to participants with long-term relationships with primary care practitioners. One participant explained that this lack of communication “doesn’t feel good at all because it’s like they’re not concerned about me, my doctors.” At times, participants also expressed worry that screening discussions could lead to uncomfortable conversations in which practitioners shame them for smoking.

## Discussion

We applied a qualitative approach to develop a conceptual framework depicting environmental, psychosocial, and modifying factors influencing lung cancer screening decisions. Study participants emphasized that community cultures influenced screening decisions, mirroring previous research suggesting that social influence is associated with screening participation.^26,28^ Our study also expands prior work^26,28^ by eliciting new insights about how aspects of the environment and society (eg, historical context) influence lung cancer screening decision-making. Participants described racism as a key factor contributing to mistrust of practitioners and avoidance of medical procedures. These findings mirror prior evidence suggesting that historical atrocities and harm, especially in Black communities, contribute to present-day mistrust of health care.^58,59^ Importantly, participants cited examples of harm they experienced in recent history (eg, having pain ignored). Previous research also found that mistrust of medical practitioners and systems arose not only from historical atrocities but also from present-day racism and negative health care experiences.^58,60,61,62,63^ To our knowledge, these findings provide some of the first evidence highlighting connections between racism and lung cancer screening decisions. Moving forward, participants noted that practitioners can increase trust by communicating effectively with patients. Such communication efforts should aim to avoid stigmatizing smoking behaviors, an issue raised by participants in our study and found in previous research.^64,65^ Our study also suggests that trust-building should entail efforts to acknowledge, understand, and reduce the present-day incidence of harmful patient experiences with practitioners. Additionally, future efforts to build trust and promote screening should meaningfully engage with communities and community-based organizations. For example, researchers, patients, and other community members might colead interventions that bring free or low-cost screening into communities through mobile units instead of relying on patients to initiate care from medical spaces that previously harmed them.

Consistent with prior literature,^21,66,67,68^ participants also highlighted psychosocial factors affecting screening decisions (eg, fear). Furthermore, participants had varying perceptions regarding their personal lung cancer risk and the importance of undergoing screening. These results point to opportunities for shared decision-making approaches that support psychosocial decision-making processes. For example, practitioners might offer information about a patient’s unique lung cancer risk along with potential benefits and screening outcomes. Additionally, shared decision-making discussions might benefit from conversations about community cultures and patient concerns regarding how their ancestors, family, and friends have historically been treated by the health care system. These discussions offer opportunities to understand concerns that would not ordinarily come up during a medical visit. For example, a practitioner might gain a new understanding of how a patient’s family member experienced harm after a cancer diagnosis and initiate conversations on ways to reduce the likelihood of similar harms occurring if the patient is diagnosed with lung cancer. Yet, prior research suggests the quality and occurrence of shared decision-making about lung cancer screening varies widely (often due to practitioner time constraints) and that these discussions often do not elicit patient values.^69,70,71,72,73,74,75^ Accordingly, future interventions are needed to promote tailored shared decision-making discussions.

Interestingly, participants identified a concern consistent with a limitation of the overall scientific evidence base: understanding whether patients who have complex health histories benefit from screening.^76,77,78^ Current USPSTF guidelines do not provide concrete guidance about LDCT for patients with comorbidities but instead note that screening should stop if the patient develops a health problem that substantially limits life expectancy or the ability or willingness to receive surgical treatment.^4^ In these situations, shared decision-making is often recommended, but more evidence is needed about how comorbidities should be incorporated into decisions.

Similar to other studies, health insurance coverage and cost were critical factors influencing decisions, even for participants with health insurance.^21^ Although Medicare and most private insurers cover LDCT in the US, Medicaid does not cover screening in all states, and patients may encounter costs for follow-up procedures.^79,80^ Participants wanted to know all potential costs up front, showcasing the need for policies that support LDCT insurance coverage and cost transparency. Participants also wanted their practitioners to have information about these costs and general screening elements (eg, screening accuracy). Yet, there is widespread variation in practitioner knowledge about lung cancer screening.^81,82,83,84^ Without such knowledge, patient-practitioner communication (a key modifying factor) may disintegrate. Novel interventions are needed to promote practitioner and patient knowledge of and meaningful communication about lung cancer screening.

### Limitations

This study has important limitations. We purposively recruited participants online, which may have excluded participants with unreliable or no internet access. Participants also self-reported information required to determine lung cancer screening eligibility (eg, smoking history), and results may have been affected by recall and other self-report biases. Additionally, most participants currently smoked, and decisions about lung cancer screening may vary among people who formerly smoked. Lastly, future community-engaged research is needed to apply and increase the transferability of our conceptual framework to larger populations.

## Conclusions

In this qualitative study of patient lung cancer screening decision-making, decisions involved interactions among environmental, psychosocial, and modifying factors. Some environmental and psychosocial factors (eg, experiencing racism and fear of a cancer diagnosis) hindered screening decisions, but intervening on modifying factors like patient-practitioner communication may offer future opportunities to facilitate screening uptake.

## Notes

Supplement 1. **eAppendix 1.** Participant Eligibility and Survey Questions **eAppendix 2.** Lung Screening Interview Guide **eAppendix 3.** Lung Screening Focus Group Guide

Supplement 2.
Data Sharing Statement
