# Supplementary material for: Patient Lung Cancer Screening Decisions and Environmental and Psychosocial Factors
Source: JAMA Netw Open. 2024 May 31;7(5):e2412880. doi: 10.1001/jamanetworkopen.2024.12880 (PMC11143466; doi:10.1001/jamanetworkopen.2024.12880)
Supplement: Supplement 1. — eAppendix 1. Participant Eligibility and Survey Questions eAppendix 2. Lung Screening Interview Guide eAppendix 3. Lung Screening Focus Group Guide [file jamanetwopen-e2412880-s001.pdf]

Supplementary Online Content

Richmond J, Fernandez JR, Bonnet K, et al. Patient lung cancer screening decisions and environmental and psychosocial factors. *JAMA Netw Open*. 2024;7(5):e2412880. doi:10.1001/jamanetworkopen.2024.12880

eAppendix 1. Participant Eligibility and Survey Questions
eAppendix 2. Lung Screening Interview Guide
eAppendix 3. Lung Screening Focus Group Guide

This supplementary material has been provided by the authors to give readers additional information about their work.

© 2024 Richmond J et al. *JAMA Network Open*.

eAppendix 1. Participant Eligibility and Survey Questions

Thank you for trying our survey! Please answer the questions starting on the next page.

Don't feel like you have to spend a long time on each question. The first answer that comes to you is usually the best one. If you aren't sure how to answer a question, choose the best answer from the options given.

© 2024 Richmond J et al. *JAMA Network Open*.

The first questions ask about basic background information.

© 2024 Richmond J et al. *JAMA Network Open*.

How old are you? Select your age in years.

- ○ 18
- ○ 19
- ○ 20
- ○ 21
- ○ 22
- ○ 23
- ○ 24
- ○ 25
- ○ 26
- ○ 27
- ○ 28
- ○ 29
- ○ 30
- ○ 31
- ○ 32
- ○ 33
- ○ 34
- ○ 35
- ○ 36
- ○ 37
- ○ 38
- ○ 39
- ○ 40
- ○ 41
- ○ 42
- ○ 43
- ○ 44
- ○ 45
- ○ 46
- ○ 47
- ○ 48
- ○ 49
- ○ 50
- ○ 51
- ○ 52
- ○ 53
- ○ 54
- ○ 55
- ○ 56
- ○ 57
- ○ 58
- ○ 59
- ○ 60
- ○ 61
- ○ 62
- ○ 63
- ○ 64
- ○ 65
- ○ 66
- ○ 67
- ○ 68
- ○ 69
- ○ 70
- ○ 71
- ○ 72
- ○ 73
- ○ 74
- ○ 75
- ○ 76
- ○ 77
- ○ 78
- ○ 79
- ○ 80
- ○ 81
- ○ 82
- ○ 83
- ○ 84
- ○

© 2024 Richmond J et al. *JAMA Network Open*.

	◯ 85<br>◯ 86<br>◯ 87<br>◯ 88<br>◯ 89<br>◯ 90<br>◯ 91<br>◯ 92<br>◯ 93<br>◯ 94<br>◯ 95<br>◯ 96<br>◯ 97<br>◯ 98<br>◯ 99
How many cigarettes have you smoked in your entire life?	◯ None, I have never smoked cigarettes<br>◯ 1-99 cigarettes (less than 5 packs)<br>◯ 100 or more cigarettes (at least 5 packs)
Do you currently smoke cigarettes?	◯ Yes<br>◯ No
How many years ago did you stop smoking cigarettes?	◯ Less than 15 years ago<br>◯ 15 years ago<br>◯ More than 15 years ago

© 2024 Richmond J et al. *JAMA Network Open*.

What age did you quit smoking for the last time?

- ◯ 1
- ◯ 2
- ◯ 3
- ◯ 4
- ◯ 5
- ◯ 6
- ◯ 7
- ◯ 8
- ◯ 9
- ◯ 10
- ◯ 11
- ◯ 12
- ◯ 13
- ◯ 14
- ◯ 15
- ◯ 16
- ◯ 17
- ◯ 18
- ◯ 19
- ◯ 20
- ◯ 21
- ◯ 22
- ◯ 23
- ◯ 24
- ◯ 25
- ◯ 26
- ◯ 27
- ◯ 28
- ◯ 29
- ◯ 30
- ◯ 31
- ◯ 32
- ◯ 33
- ◯ 34
- ◯ 35
- ◯ 36
- ◯ 37
- ◯ 38
- ◯ 39
- ◯ 40
- ◯ 41
- ◯ 42
- ◯ 43
- ◯ 44
- ◯ 45
- ◯ 46
- ◯ 47
- ◯ 48
- ◯ 49
- ◯ 50
- ◯ 51
- ◯ 52
- ◯ 53
- ◯ 54
- ◯ 55
- ◯ 56
- ◯ 57
- ◯ 58
- ◯ 59
- ◯ 60

© 2024 Richmond J et al. *JAMA Network Open*.

- ◯ 61
- ◯ 62
- ◯ 63
- ◯ 64
- ◯ 65
- ◯ 66
- ◯ 67
- ◯ 68
- ◯ 69
- ◯ 70
- ◯ 71
- ◯ 72
- ◯ 73
- ◯ 74
- ◯ 75
- ◯ 76
- ◯ 77
- ◯ 78
- ◯ 79
- ◯ 80

© 2024 Richmond J et al. *JAMA Network Open*.

What age did you start smoking cigarettes?

- ◯ 1
- ◯ 2
- ◯ 3
- ◯ 4
- ◯ 5
- ◯ 6
- ◯ 7
- ◯ 8
- ◯ 9
- ◯ 10
- ◯ 11
- ◯ 12
- ◯ 13
- ◯ 14
- ◯ 15
- ◯ 16
- ◯ 17
- ◯ 18
- ◯ 19
- ◯ 20
- ◯ 21
- ◯ 22
- ◯ 23
- ◯ 24
- ◯ 25
- ◯ 26
- ◯ 27
- ◯ 28
- ◯ 29
- ◯ 30
- ◯ 31
- ◯ 32
- ◯ 33
- ◯ 34
- ◯ 35
- ◯ 36
- ◯ 37
- ◯ 38
- ◯ 39
- ◯ 40
- ◯ 41
- ◯ 42
- ◯ 43
- ◯ 44
- ◯ 45
- ◯ 46
- ◯ 47
- ◯ 48
- ◯ 49
- ◯ 50
- ◯ 51
- ◯ 52
- ◯ 53
- ◯ 54
- ◯ 55
- ◯ 56
- ◯ 57
- ◯ 58
- ◯ 59
- ◯ 60
- ◯

© 2024 Richmond J et al. *JAMA Network Open*.

	◯ 61<br>◯ 62<br>◯ 63<br>◯ 64<br>◯ 65<br>◯ 66<br>◯ 67<br>◯ 68<br>◯ 69<br>◯ 70<br>◯ 71<br>◯ 72<br>◯ 73<br>◯ 74<br>◯ 75<br>◯ 76<br>◯ 77<br>◯ 78<br>◯ 79<br>◯ 80
Total years of smoking for current smokers	________________________
Total years of smoking for former smokers	________________________
About how many cigarettes do you currently smoke per day? There are 20 cigarettes in a pack.	◯ Less than a quarter pack<br>◯ Quarter pack<br>◯ Half pack<br>◯ One pack<br>◯ One and a half packs<br>◯ Two packs<br>◯ Two and half packs<br>◯ Three packs<br>◯ Three and a half packs<br>◯ Four packs<br>◯ Four and a half packs<br>◯ Five packs<br>◯ Six packs or more
Number of pack-years for current smokers	________________________
About how many cigarettes did you previously smoke per day? There are 20 cigarettes in a pack.	◯ Less than a quarter pack<br>◯ Quarter pack<br>◯ Half pack<br>◯ One pack<br>◯ One and a half packs<br>◯ Two packs<br>◯ Two and half packs<br>◯ Three packs<br>◯ Three and a half packs<br>◯ Four packs<br>◯ Four and a half packs<br>◯ Five packs<br>◯ Six packs or more
Number of pack-years for former smokers	________________________

© 2024 Richmond J et al. *JAMA Network Open*.

Has a doctor ever told you that you have lung cancer?	◯ Yes<br>◯ No
Are you comfortable reading and talking in English?	◯ Yes<br>◯ No

© 2024 Richmond J et al. *JAMA Network Open*.

Which state do you currently reside in?	◯ I do not live in the U.S.<br>◯ Alabama<br>◯ Alaska<br>◯ Arizona<br>◯ Arkansas<br>◯ California<br>◯ Colorado<br>◯ Connecticut<br>◯ Delaware<br>◯ Florida<br>◯ Georgia<br>◯ Hawaii<br>◯ Idaho<br>◯ Illinois<br>◯ Indiana<br>◯ Iowa<br>◯ Kansas<br>◯ Kentucky<br>◯ Louisiana<br>◯ Maine<br>◯ Maryland<br>◯ Massachusetts<br>◯ Michigan<br>◯ Minnesota<br>◯ Mississippi<br>◯ Missouri<br>◯ Montana<br>◯ Nebraska<br>◯ Nevada<br>◯ New Hampshire<br>◯ New Jersey<br>◯ New Mexico<br>◯ New York<br>◯ North Carolina<br>◯ North Dakota<br>◯ Ohio<br>◯ Oklahoma<br>◯ Oregon<br>◯ Pennsylvania<br>◯ Rhode Island<br>◯ South Carolina<br>◯ South Dakota<br>◯ Tennessee<br>◯ Texas<br>◯ Utah<br>◯ Vermont<br>◯ Virginia<br>◯ Washington<br>◯ West Virginia<br>◯ Wisconsin<br>◯ Wyoming<br>◯ District of Columbia

Please click "Submit" to continue.

© 2024 Richmond J et al. *JAMA Network Open*.

Have you heard that lung cancer screening is recommended for some people who smoke and are at high risk?	◯ Yes<br>◯ No
Have you ever had a test to look for early lung cancer?	◯ Yes<br>◯ No<br>◯ I don't know
What test did you have to look for early lung cancer?	◯ Chest X-ray<br>◯ Chest CT (CAT) scan<br>◯ Chest MRI<br>◯ Blood test<br>◯ Other<br>◯ Unsure/don't know
Please describe the test you had to look for early lung cancer.	______________________________
Which of the following statements best describes your family history of lung cancer?	◯ At least one first degree relative (mother, father, brother, sister not including step-relatives) had lung cancer<br>◯ At least one second degree relative (cousins, grandparents) had lung cancer<br>◯ I have no family history of lung cancer<br>◯ I am unsure/don't know
In the past, have you ever made a serious attempt to quit smoking? That is, have you stopped smoking for at least one day or longer because you were trying to quit?	◯ Yes<br>◯ No
How soon after you wake up do you smoke your first cigarette?	◯ Within 5 minutes<br>◯ 6 to 30 minutes<br>◯ 31 to 60 minutes<br>◯ After 60 minutes
When you smoked cigarettes, how soon after you woke up did you smoke your first cigarette?	◯ Within 5 minutes<br>◯ 6 to 30 minutes<br>◯ 31 to 60 minutes<br>◯ After 60 minutes
What sex were you assigned at birth, on your original birth certificate?	◯ Male<br>◯ Female
How do you describe your gender identity?	◯ Male<br>◯ Female<br>◯ Non-binary<br>◯ A gender identity not listed here
How do you describe your gender?	______________________________

© 2024 Richmond J et al. *JAMA Network Open*.

Which categories describe you? Select all that apply. Note, you may select more than one group.

☐ Black, African American or African (For example: African American, Ethiopian, Haitian, Jamaican, Nigerian, Somali, etc.)
☐ White (For example: English, European, French, German, Irish, Italian, Polish, etc.)
☐ Hispanic, Hispanic, Latino, or Spanish (For example: Columbian, Cuban, Dominican, Mexican or Mexican American, Puerto Rican, Salvadoran, etc.)
☐ American Indian or Alaska Native (For example: Aztec, Blackfeet Tribe, Mayan, Navajo Nation, Native Village of Barrow Inupiat Traditional Government, Nome Eskimo Community, etc.)
☐ Asian (For example: Asian Indian, Chinese, Filipino, Japanese, Korean, Vietnamese, etc.)
☐ Middle Eastern or North African (For example: Algerian, Egyptian, Iranian, Lebanese, Moroccan, Syrian, etc.)
☐ Native Hawaiian or other Pacific Islander (For example: Chamorro, Fijian, Marshallese, Native Hawaiian, Tongan, etc.)
☐ None of these fully describe me

Please specify.

____

American Indian or Alaska Native (Select all that apply)

☐ American Indian
☐ Alaska Native
☐ Central or South American Indian
☐ None of these fully describe me

Provide the name of the tribe in which you are enrolled or affiliated or your tribal descent. (for example, Navajo Nation, Blackfeet Tribe, Mayan, Aztec, Native Village of Barrow Inupiat Traditional Government, Nome Eskimo Community, etc.)

____

Please specify.

____

Asian (Select all that apply)

☐ Asian Indian
☐ Cambodian
☐ Chinese
☐ Filipino
☐ Hmong
☐ Japanese
☐ Korean
☐ Pakistani
☐ Vietnamese
☐ None of these fully describe me

Please specify.

____

© 2024 Richmond J et al. *JAMA Network Open*.

Black, African American or African (Select all that apply)

☐ African American
☐ Barbadian
☐ Caribbean
☐ Ethiopian
☐ Ghanaian
☐ Haitian
☐ Jamaican
☐ Liberian
☐ Nigerian
☐ Somali
☐ South African
☐ None of these fully describe me

Please specify.

____

Hispanic, Latino, or Spanish (Select all that apply)

☐ Colombian
☐ Cuban
☐ Dominican
☐ Ecuadorian
☐ Honduran
☐ Mexican or Mexican American
☐ Puerto Rican
☐ Salvadoran
☐ Spanish
☐ None of these fully describe me

Please specify.

____

Middle Eastern or North African (Select all that apply)

☐ Afghan
☐ Algerian
☐
Egyptian
☐ Iranian
☐ Iraqi
☐ Israeli
☐ Lebanese
☐ Moroccan
☐ Syrian
☐ Tunisian
☐ None of these fully describe me

Please specify.

____

© 2024 Richmond J et al. *JAMA Network Open*.

Native Hawaiian or other Pacific Islander (Select all that apply)

☐ Chamorro
☐ Chuukese
☐ Fijian
☐ Marshallese
☐ Native
☐ Hawaiian
☐ Palauan
☐ Samoan
Tahitian
☐ Tongan
☐ None of these fully describe me

Please specify.

____

White (Select all that apply)

☐ Dutch
☐ English
☐ European
☐ French
☐ German
☐ Irish
☐ Italian
☐ Norwegian
☐ Polish
☐ Scottish
☐ Spanish
☐ None of these fully describe me

Please specify.

____

What is the highest degree or level of school you have completed?

◯ Less Than High School Degree
◯ High School Graduate
◯ GED
◯ Some College or Technical School
◯ Associate's Degree (2-year)
◯ Bachelor's Degree (4-year)
◯ Master's Degree
◯ Doctoral Degree (PhD)
◯ Professional Degree (JD, MD)

What is your current marital status?

◯ Never Married
◯ Married or Domestic Partnership
◯ Living with a Partner
◯ Separated
◯ Divorced
◯ Widowed

© 2024 Richmond J et al. *JAMA Network Open*.

Would you please give your best guess for your household income?<br><br>Please indicate the answer that includes your entire household income in the last 12 months (previous year) before taxes.	◯ Less than $10,000<br>◯ $10,000 to $19,999<br>◯ $20,000 to $29,999<br>◯ $30,000 to $39,999<br>◯ $40,000 to $49,999<br>◯ $50,000 to $59,999<br>◯ $60,000 to $69,999<br>◯ $70,000 to $79,999<br>◯ $80,000 to $89,999<br>◯ $90,000 to $99,999<br>◯ $100,000 to $149,999<br>◯ $150,000 or More
How many people live in your household, including yourself?	◯ 1 person<br>◯ 2 people<br>◯ 3 people<br>◯ 4 people<br>◯ 5 people<br>◯ 6 people<br>◯ 7 people<br>◯ 8 or more people
Are you currently...?<br><br>If more than one category fits, select the category which best describes you.	◯ Employed full-time<br>◯ Employed part-time<br>◯ Unemployed and currently seeking employment<br>◯ Unemployed and not seeking employment<br>◯ A student<br>◯ Retired<br>◯ Unable to Work<br>◯ Other
Please specify your employment status.	________________________
What is the primary source of your health care insurance/coverage? Is it...	◯ A plan purchased through an employer or union (This includes plans purchased through another person's employer)<br>◯ A plan that you or another family member buys on your own<br>◯ Medicare<br>◯ Medicaid or other state program<br>◯ TRICARE (formerly CHAMPUS), VA, or<br>◯ Military Alaska Native, Indian Health Service, Tribal Health Services<br>◯ Some other source<br>◯ None (no coverage)
Please specify the source of your health care insurance/coverage.	________________________
Do you have one person you think of as your personal doctor or health care provider?	◯ Yes<br>◯ No
Where do you receive most of your medical care?	◯ Private Physician's Office<br>◯ Hospital Outpatient Clinic<br>◯ Clinic or Health Center<br>◯ Emergency Room<br>◯ Other

© 2024 Richmond J et al. *JAMA Network Open*.

Please specify where you receive most of your medical care.	______________________________

© 2024 Richmond J et al. *JAMA Network Open*.

eAppendix 2. Lung Screening Interview Guide

Instructions
Thanks for joining me today to participate in this interview. [Interviewer introduces self].

Dr. Jennifer Richmond is working on a research study to understand community perspectives on lung screening. We would like to know your thoughts about how we can help more people receive a lung screening test, which can help catch lung cancer earlier when it is easier to treat.

Before we get started, I would like to provide some background information about our study. [Review the information sheet with participant, including benefits, risks, information about protecting privacy, and consent for audio-recording].

Questions for participants who have previously received lung screening

Lung screening is important for people who smoke or who have quit smoking less than 15 years ago. Lung screening is a procedure that can detect lung cancer early. When detected early, lung cancer is less severe and less likely to lead to death. The number of people who take advantage of lung screening is low. One of the goals of our research is to increase the number of people who are screened. We want to talk to you today because you have had the experience of lung screening. We are interested in your thoughts and feelings about your experiences with lung screening.

1. How did you first find out about lung screening?
    a. Who first mentioned lung screening to you (a doctor, nurse, friend, etc.)?
2. When did you talk about lung screening with your doctor?
3. What was your initial reaction to the idea of scheduling a lung screening?
    a. What concerns did you have about it?
    b. What did you see as the potential benefits of lung screening?
    c. What did you see as the potential harms of lung screening?
4. Walk me through how you made the decision to schedule a lung screening.
    a. What discussion, if any, did you have with your doctor about lung screening?
    b. What did you do to learn more about lung screening before making your decision?
    c. What were the most useful sources of information for you?
        i. In what ways were those useful?
        ii. What information was the least useful?
    d. Why did you decide to have the screening?
5. Tell me about your experience receiving lung screening.
    a. What did you have to do to schedule the appointment? About how long did it take you to get an appointment scheduled?
    b. What transportation did you use to get to the appointment?
    c. How long did you have to wait at the clinic?
    d. How much did you have to pay out of pocket for the lung screening?
    e. How were the results given to you? How long did it take to receive the results?
        i. Did anyone explain the meaning of the results to you?
        ii. How did you feel while waiting for the results? How did you feel once you received the results?
        iii. Did you share the lung screening results with friends of family? If so, tell me about that experience. When you receive results from other medical tests, do you typically share them with friends of family? [probe to understand how they share results and what this experience is like. If they do not share results, probe to understand why]
    f. What recommendations, if any, were made for follow up?
6. What things went well during your lung screening experience? What parts of the experience were negative or unpleasant?
7. We are trying to understand how we can make this experience better. What things could have been changed to make your lung screening experience more positive?
8. What are some reasons why someone you know who is eligible might *not* get screened for lung cancer? (Probe to understand barriers, such as fear, stigma, medical mistrust, concerns about cost, transportation issues, etc.)
    a. What are some things that could be done to address these issues?
9. Researchers have found that some patients may not get screened because they do not trust the health care system and/or doctors. Why do you think that is?
    a. Has mistrust in the healthcare system or doctors been a concern of yours? If so, in what way?

© 2024 Richmond J et al. *JAMA Network Open*.

b. How much is mistrust and trust in the health system an issue for other members of your family? Other people you know in your community? (Probe to understand how participant defines "community").
c. How do you know whether or not you can trust a doctor? What about nurses or other health care providers?
    i. Probe to understand factors that make them trust or mistrust doctors/health care providers (e.g., negative experiences, Tuskegee).
    ii. How do your past experiences with doctors and health care providers impact how much you trust health care providers?
    iii. What makes doctors and other health care providers seem trustworthy?
d. How would you define "mistrust" of the health care system or doctors? What does it look like when someone mistrusts doctors?
e. How would you define "trust" in the health care system or doctors? What does it look like when someone trust doctors?
f. How might mistrust affect someone's decision to get screened for lung cancer?
g. What can we do to help patients who do not trust the health system and/or doctors to get screened for lung cancer?

10. What are the best ways to educate people about getting screened?
    a. What messages do you think would help people who are hesitant to have lung screening decide to schedule an appointment?
    b. Which is more important, emphasizing the benefits of screening or emphasizing the risks of not being screened?
    c. We've heard from other people that words matter a lot when thinking about cancer screening messages. Some people prefer to think about cancer using terms like battle, fight, and enemy. Other people prefer to think about cancer using terms like journey, travel, and path. Which way do you prefer to think about cancer? Why? How, if at all, would these messages affect your willingness to get screened?
        i. If participant asks for an example:
            1. Battle: Lung cancer screening can help us win the battle against cancer.
            2. Journey: Lung screening is a tool that can help us on the journey to catch cancer early.
    d. What do you think are the best ways to spread the word / raise awareness about lung screening to people in your community?
11. What else do you have to say about cancer screening in general or lung cancer screening in particular?

Questions for participants who are eligible but have <u>not been screened</u>

Lung screening is important for people who smoke or who have quit smoking less than 15 years ago. Lung screening is a procedure that can detect lung cancer early. When detected early, lung cancer is less severe and less likely to lead to death. The number of people who take advantage of lung screening is low. One of the goals of our research is to increase the number of people who are screened. We are interested in your thoughts and feelings about the idea of lung screening. There are no right or wrong answers. We just want to learn your thoughts and opinions about lung screening.

1. What has your experience been getting screened for any health condition? (e.g., colonoscopy, diabetes)
    a. Describe any positive experiences you have had with health screening?
    b. Describe any negative or unpleasant experience you have had with health screening?
2. What do you know about cancer screening?
    a. What are your past experiences with cancer screening, both positive and negative?
3. How do people you know talk about cancer (e.g., openly, do not discuss)?
    a. How much of a concern is cancer to you? To your family? To your community? (Probe to understand how participant defines/describes "community")
    b. How comfortable are you talking about cancer? How comfortable are friends and family members talking about cancer?
    c. What about cancer screening? How important is cancer screening to the people you know?
4. Researchers have found that some patients may not get screened because they do not trust the health care system and/or doctors. Why do you think that is?
    a. Has mistrust in the healthcare system or doctors been a concern of yours? If so, in what way?
    b. How much is mistrust and trust in the health system an issue for other members of your family? Other people you know in your community? (Probe to understand how participant defines "community").
    c. How do you know whether or not you can trust a doctor? What about nurses or other health care providers?
        i. Probe to understand factors that make them trust or mistrust doctors/health care providers (e.g., negative experiences, Tuskegee).

© 2024 Richmond J et al. *JAMA Network Open*.

ii. How do your past experiences with doctors and health care providers impact how much you trust health care providers?
iii. What makes doctors and other health care providers seem trustworthy?

d. How would you define “mistrust” of the health care system or doctors? What does it look like when someone mistrusts doctors?
e. How would you define “trust” in the health care system or doctors? What does it look like when someone trust doctors?
f. How might mistrust affect someone’s decision to get screened for lung cancer?
g. What can we do to help patients who do not trust the health system and/or doctors to get screened for lung cancer?

For the rest of this interview, I’ll be asking specific questions about screening for lung cancer.

Provide Background Information to Participants

I’m now going to provide some background information about lung cancer. You may already know this information, but we are going over this information with study participants so that we are all on the same page.

Lung cancer is one of the most common cancers diagnosed in men and women in the US. Doctors can look for signs of lung cancer before symptoms occur using a test called low-dose computed tomography. The test is described as low-dose because the amount of radiation is low compared to regular scans or x-rays. The patient does not have to drink any fluid or have injections. During a lung screening exam, you lie on your back on a long table. You would be asked to lie still while the table slides through a machine that takes an image of your lungs. The scan takes less than a minute. After the scan is complete, you can go about your day normally. As your body is scanned, a computer creates images of your lungs. Doctors look at these images for anything that might be a sign of lung cancer. Receiving this procedure is called lung screening. Lung screening can help doctors find lung cancer early when it is easier to treat. People are eligible for lung screening if they are between the ages of 50-80 and have a history of heavy tobacco use. People are eligible if they currently smoke or have quit smoking within the past 15 years.
Today, we are interested in learning about your thoughts and feelings about lung screening. We hope to learn ways to help more people who are eligible get screened.

5. Now that I’ve described screening for lung cancer, what questions do you have?
   i. Any other questions you have?
   ii. Are there questions you might ask a doctor about lung screening?
6. Based on what I’ve told you, do you think you are eligible for lung cancer screening? Why or why not?
   a. If participant thinks they are eligible:
      i. How likely is it that you would agree to be screened for lung cancer? Why or why not?
      ii. What reasons might make you want to get screened for lung cancer? (Probe to understand benefits, such as early detection of cancer, peace of mind, etc.)
   b. If participant does not think they are eligible:
      i. For someone who is eligible, what do you think are the reasons to get screened? (Probe to understand benefits, such as early detection of cancer, peace of mind, etc.)
7. What are some reasons why you or someone you know who is eligible might *not* get screened for lung cancer? (Probe to understand barriers, such as fear, stigma, medical mistrust, concerns about cost, etc.)
8. What information would you need to decide about lung screening?
   a. What sources would you trust for this information (e.g., doctors, friends, a pastor, the internet)?
   b. What concerns do you have about lung screening?
      i. Note: If participant brings up harms of screening (e.g., radiation), follow up to understand these concerns and how, if at all, they might be alleviated.
9. What else do you have to say about cancer screening in general or lung cancer screening in particular?

© 2024 Richmond J et al. *JAMA Network Open*.

eAppendix 3. Lung Screening Focus Group Guide

Instructions

Thank you all for joining me today to participate in this focus group. [Facilitator introduces self].

Dr. Jennifer Richmond is working on a research study to understand community perspectives on lung screening. We would like to know your thoughts about how we can help more people receive a lung screening test, which can help catch lung cancer earlier when it is easier to treat.

Before we get started, I would like to provide some background information about our study. [Review the information sheet with participants, including benefits, risks, information about protecting privacy, and consent for audio-recording].

Questions for participants who have previously received lung screening

Lung screening is important for people who smoke or who have quit smoking less than 15 years ago. Lung screening is a procedure that can detect lung cancer early. When detected early, lung cancer is less severe and less likely to lead to death. The number of people who take advantage of lung screening is low. One of the goals of our research is to increase the number of people who are screened. We want to talk to you today because you have had the experience of lung screening. We are interested in your thoughts and feelings about your experiences with lung screening.

1. How did you first find out about lung screening?
   a. Who first mentioned lung screening to you (a doctor, nurse, friend, etc.)?
2. When did you talk about lung screening with your doctor?
3. What was your initial reaction to the idea of scheduling a lung screening?
   a. What concerns did you have about it?
   b. What did you see as the potential benefits of lung screening?
   c. What did you see as the potential harms of lung screening?
4. How did you made the decision to schedule a lung screening?
   a. What discussion, if any, did you have with your doctor about lung screening?
   b. What did you do to learn more about lung screening before making your decision?
   c. What were the most useful sources of information for you?
      i. In what ways were those useful?
      ii. What information was the least useful?
   d. Why did you decide to have the screening?
5. Tell me about your experience receiving lung screening.
   a. What did you have to do to schedule the appointment? About how long did it take you to get an appointment scheduled?
   b. What transportation did you use to get to the appointment?
   c. How long did you have to wait at the clinic?
   d. How much did you have to pay out of pocket for the lung screening?
   e. How were the results given to you? How long did it take to receive the results?
      i. Did anyone explain the meaning of the results to you?
      ii. How did you feel while waiting for the results? How did you feel once you received the results?
      iii. Did you share the lung screening results with friends of family? If so, tell me about that experience. When you receive results from other medical tests, do you typically share them with friends of family? [probe to understand how they share results and what this experience is like. If they do not share results, probe to understand why]
   f. What recommendations, if any, were made for follow up?
6. What things went well during your lung screening experience? What parts of the experience were negative or unpleasant?
7. We are trying to understand how we can make this experience better. What things could have been changed to make your lung screening experience more positive?
8. What are some reasons why someone you know who is eligible might *not* get screened for lung cancer? (Probe to understand barriers, such as fear, stigma, medical mistrust, concerns about cost, transportation issues, etc.)
   a. What are some things that could be done to address these issues?

© 2024 Richmond J et al. *JAMA Network Open*.

9. Researchers have found that some patients may not get screened because they do not trust the health care system and/or doctors. Why do you think that is?
    a. Has mistrust in the healthcare system or doctors been a concern of yours? If so, in what way?
    b. How much is mistrust and trust in the health system an issue for other members of your family? Other people you know in your community? (Probe to understand how participants define "community").
    c. How do you know whether or not you can trust a doctor? What about nurses or other health care providers?
        i. Probe to understand factors that make them trust or mistrust doctors/health care providers (e.g., negative experiences, Tuskegee).
        ii. How do your past experiences with doctors and health care providers impact how much you trust health care providers?
        iii. What makes doctors and other health care providers seem trustworthy?
    d. How would you define "mistrust" of the health care system or doctors? What does it look like when someone mistrusts doctors?
    e. How would you define "trust" in the health care system or doctors? What does it look like when someone trust doctors?
    f. How might mistrust affect someone's decision to get screened for lung cancer?
    g. What can we do to help patients who do not trust the health system and/or doctors to get screened for lung cancer?
10. What are the best ways to educate people about getting screened?
    a. What messages do you think would help people who are hesitant to have lung screening decide to schedule an appointment?
    b. Which is more important, emphasizing the benefits of screening or emphasizing the risks of not being screened?
    c. We've heard from other people that words matter a lot when thinking about cancer screening messages. Some people prefer to think about cancer using terms like battle, fight, and enemy. Other people prefer to think about cancer using terms like journey, travel, and path. Which way do you prefer to think about cancer? Why? How, if at all, would these messages affect your willingness to get screened?
        i. If participant asks for an example:
            1. Battle: Lung cancer screening can help us win the battle against cancer.
            2. Journey: Lung screening is a tool that can help us on the journey to catch cancer early.
    d. What do you think are the best ways to spread the word / raise awareness about lung screening to people in your community?
11. What else do you have to say about cancer screening in general or lung cancer screening in particular?

Questions for participants who are eligible but have <u>not been screened</u>

Lung screening is important for people who smoke or who have quit smoking less than 15 years ago. Lung screening is a procedure that can detect lung cancer early. When detected early, lung cancer is less severe and less likely to lead to death. The number of people who take advantage of lung screening is low. One of the goals of our research is to increase the number of people who are screened. We are interested in your thoughts and feelings about the idea of lung screening. There are no right or wrong answers. We just want to learn your thoughts and opinions about lung screening.

1. What has your experience been getting screened for any health condition? (e.g., colonoscopy, diabetes)
    a. Describe any positive experiences you have had with health screening?
    b. Describe any negative or unpleasant experience you have had with health screening?
2. What do you know about cancer screening?
    a. What are your past experiences with cancer screening, both positive and negative?
3. How do people you know talk about cancer (e.g., openly, do not discuss)?
    a. How much of a concern is cancer to you? To your family? To your community? (Probe to understand how participant defines/describes "community")
    b. How comfortable are you talking about cancer? How comfortable are friends and family members talking about cancer?
    c. What about cancer screening? How important is cancer screening to the people you know?

© 2024 Richmond J et al. *JAMA Network Open*.

4. Researchers have found that some patients may not get screened because they do not trust the health care system and/or doctors. Why do you think that is?
    a. Has mistrust in the healthcare system or doctors been a concern of yours? If so, in what way?
    b. How much is mistrust and trust in the health system an issue for other members of your family? Other people you know in your community? (Probe to understand how participants define “community”).
    c. How do you know whether or not you can trust a doctor? What about nurses or other health care providers?
        i. Probe to understand factors that make them trust or mistrust doctors/health care providers (e.g., negative experiences, Tuskegee).
        ii. How do your past experiences with doctors and health care providers impact how much you trust health care providers?
        iii. What makes doctors and other health care providers seem trustworthy?
    d. How would you define “mistrust” of the health care system or doctors? What does it look like when someone mistrusts doctors?
    e. How would you define “trust” in the health care system or doctors? What does it look like when someone trust doctors?
    f. How might mistrust affect someone’s decision to get screened for lung cancer?
    g. What can we do to help patients who do not trust the health system and/or doctors to get screened for lung cancer?

For the rest of this focus group, I’ll be asking specific questions about screening for lung cancer.

Provide Background Information to Participants

I’m now going to provide some background information about lung cancer. You may already know this information, but we are going over this information with study participants so that we are all on the same page.

Lung cancer is one of the most common cancers diagnosed in men and women in the US. Doctors can look for signs of lung cancer before symptoms occur using a test called low-dose computed tomography. The test is described as low-dose because the amount of radiation is low compared to regular scans or x-rays. The patient does not have to drink any fluid or have injections. During a lung screening exam, you lie on your back on a long table. You would be asked to lie still while the table slides through a machine that takes an image of your lungs. The scan takes less than a minute. After the scan is complete, you can go about your day normally. As your body is scanned, a computer creates images of your lungs. Doctors look at these images for anything that might be a sign of lung cancer.

Receiving this procedure is called lung screening. Lung screening can help doctors find lung cancer early when it is easier to treat. People are eligible for lung screening if they are between the ages of 50-80 and have a history of heavy tobacco use. People are eligible if they currently smoke or have quit smoking within the past 15 years.

Today, we are interested in learning about your thoughts and feelings about lung screening. We hope to learn ways to help more people who are eligible get screened.

5. Now that I’ve described screening for lung cancer, what questions do you have?
        i. Any other questions you have?
        ii. Are there questions you might ask a doctor about lung screening?
6. Based on what I’ve told you, how likely is it that you would agree to be screened for lung cancer? Why or why not?
        i. What reasons might make you want to get screened for lung cancer? (Probe to understand benefits, such as early detection of cancer, peace of mind, etc.)
    b. If some participants do not think they are eligible:
        i. For someone who is eligible, what do you think are the reasons to get screened? (Probe to understand benefits, such as early detection of cancer, peace of mind, etc.)
7. What are some reasons why you or someone you know who is eligible might *not* get screened for lung cancer? (Probe to understand barriers, such as fear, stigma, medical mistrust, concerns about cost, etc.)
8. What information would you need to decide about lung screening?
    c. What sources would you trust for this information (e.g., doctors, friends, a pastor, the internet)?
    d. What concerns do you have about lung screening?
        i. Note: If participant brings up harms of screening (e.g., radiation), follow up to understand these concerns and how, if at all, they might be alleviated.
9. What else do you have to say about cancer screening in general or lung cancer screening in particular?

© 2024 Richmond J et al. *JAMA Network Open*.
