# Supplementary material for: Patient Lung Cancer Screening Decisions and Environmental and Psychosocial Factors
Source: JAMA Netw Open. 2024 May 31;7(5):e2412880. doi: 10.1001/jamanetworkopen.2024.12880 (PMC11143466; doi:10.1001/jamanetworkopen.2024.12880)
Supplement: Supplement 2. — Data Sharing Statement [file jamanetwopen-e2412880-s002.pdf]

Data Sharing Statement

Richmond. Patient Lung Cancer Screening Decisions and Environmental and Psychosocial Factors. *JAMA Netw Open*. Published May 31, 2024.
doi:10.1001/jamanetworkopen.2024.12880

Data
Data available: No

Additional Information
Explanation for why data not available: To avoid sharing identifying participant information, the data from this qualitative study (i.e., transcripts of interviews and focus groups) are not publicly available. However, qualified researchers may contact the corresponding author to inquire about data sharing once a data use agreement is in place.
